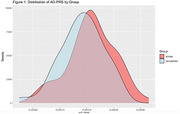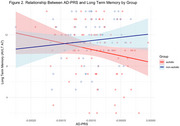# Alzheimer's disease genetic risk dosage and verbal memory in autistic adults

**DOI:** 10.1002/alz70860_100506

**Published:** 2025-12-23

**Authors:** Samantha A Harker, Ignazio Piras, Matthew J Huentelman, Francis Taguinod, Candace R Lewis, B. Blair Braden

**Affiliations:** ^1^ Arizona State University, Tempe, AZ, USA; ^2^ The Translational Genomics Research Institute (TGen‐ an Affiliate of City of Hope), Phoenix, AZ, USA

## Abstract

**Background:**

Recent evidence from Medicare data suggests that older autistic adults are 18 times more likely to be diagnosed with Alzheimer's disease (AD) compared to non‐autistic adults but understanding of heterogeneity in cognitive and brain aging with autism is lacking. Genetics explain substantial variance in cognitive aging outcomes and incidence of neurodegenerative disease, but there are no genetically‐informed studies of aging with autism. Polygenic risk scores (PRS) are a tool to measure an individual's genetic liability to a certain trait, which has shown to be very effective for predicting AD cases. We aim to 1) Determine whether autistic individuals carry a higher cumulative genetic risk for AD compared to non‐autistic individuals and 2) Determine if AD‐PRS differentially moderates memory performance in autistic vs. non‐autistic adults

**Method:**

Participants were 53 autistic and 48 non‐autistic adults ages 18‐70 (38.62±16.75). DNA was extracted from saliva using Oragene's DNA purification protocol and genotyped on the Illumina Global Diversity Array with an additional 180K neurodegenerative disease markers, then imputed for whole genome coverage through the TOPMed server. AD‐PRS was generated from a Genome Wide Association Study of 71,880 AD and 383,378 controls using the PRSice‐2 software. We performed an ANCOVA to test a diagnostic group difference in AD‐PRS, controlling for sex, and the interaction between group and sex. Additionally, we tested a diagnostic group difference in long‐term memory (AVLT A7), controlling for sex, age, AD‐PRS, and the interaction between group, sex, and AD‐PRS.

**Result:**

The autism group had a significantly larger AD‐PRS (*p* = 0.030), and significantly worse long‐term memory scores (*p* = 0.043) than non‐autistic controls. There was an interaction between autism diagnosis and AD‐PRS (*p* = 0.05) that was driven by a negative slope in the autism group.

**Conclusion:**

This study was the first to investigate cumulative genetic risk for AD in autistic adults and found that autistic adults have a greater genetic risk in comparison to non‐autistic adults. Synoptically, we found that the Alzheimer's PRS has a greater negative impact on cognitive function in autistic adults compared to non‐autistic adults.